# Exploring Emerging Trends in Climate Change’s Impacts on the Cardiopulmonary Health of Adults Living in the Canton of Valais, Switzerland: Preliminary Autumn and Winter Results from a Pilot Study

**DOI:** 10.3390/ijerph23020274

**Published:** 2026-02-23

**Authors:** Omar Portela Dos Santos, Florence Selz Amaudruz, Paulo Jorge Pereira Alves, Henk Verloo

**Affiliations:** 1 Department of Nursing Sciences, School of Health Sciences, HES-SO Valais/Wallis, 1950 Sion, Switzerland; henk.verloo@hevs.ch; 2 Faculty of Health Sciences and Nursing, Catholic University of Portugal, Rua de Diogo Botelho 1327, 4169-005 Porto, Portugal; pjalves@ucp.pt; 3 Emergency Department, Sion Hospital, Valais Romand Hospital Center, 1951 Sion, Switzerland; florence.selzamaudruz@hopitalvs.ch; 4 Centre for Interdisciplinary Research in Health (CIIS—Wounds Research Lab), Universidade Católica Portuguesa, 4169-005 Porto, Portugal; 5 Service of Old Age Psychiatry, Department of Psychiatry, Lausanne University Hospital, 1011 Lausanne, Switzerland

**Keywords:** climate change, global warming, public health issues, environment and public health, health literacy, information literacy, eco-literacy, nurses, cardiopulmonary diseases, emergency department consultations

## Abstract

**Highlights:**

**Public health relevance—How does this work relate to a public health issue?**

- This study provides preliminary evidence that short-term variations in temperature and air pollution are associated with fluctuations in cardiopulmonary emergency department admissions, highlighting the vulnerability of aging and chronically ill populations in climate-sensitive alpine regions.
- By demonstrating the feasibility of linking real-time environmental data with healthcare utilization, this work informs public health surveillance, early-warning systems, and climate-resilient healthcare planning aimed at anticipating demand for emergency care and reducing preventable morbidity under changing climatic conditions.

**Public health significance—Why is this work of significance to public health?**

- This study strengthens the evidence base on how environmental exposures related to climate change influence emergency department demand, thereby supporting population-level risk assessment and informing strategies to protect vulnerable groups from climate-related health impacts.
- Its significance lies in its potential to support proactive public health action by integrating environmental and health system data, enabling earlier detection of climate-sensitive health effects and guiding preventive, adaptive, and resource-allocation strategies within healthcare systems.

**Public health implications—What are the key implications or messages for practitioners, policy makers and/or researchers in public health?**

- For public health practitioners and policymakers, these findings underscore the need to integrate environmental and climatic indicators into routine health surveillance and emergency preparedness planning to anticipate climate-sensitive fluctuations in healthcare.
- For researchers, this study highlights the value of interdisciplinary, data-linkage approaches combining environmental monitoring and health service utilization, supporting further longitudinal and multi-site research to inform evidence-based climate adaptation strategies within health systems.

**Abstract:**

Background: Climate change and air pollution are major threats to cardiopulmonary health, yet their population-level impacts in alpine regions remain insufficiently documented. Methods: This pilot study aimed to generate preliminary evidence and assess the feasibility of a larger investigation by examining associations between meteorological and air pollution variables and adult cardiopulmonary emergency department admissions in the canton of Valais, Switzerland. Results: Weekly admissions averaged 4.2 cases (range: 1–14), with peaks in late January and early February. Mean weekly temperature was inversely associated with admissions (IRR = 0.92), indicating higher demand during colder weeks. Ozone exposure showed a positive but non-statistically significant association with weekly cardiopulmonary admissions (IRR = 1.014), suggesting a potential signal that warrants confirmation in larger studies. A demographic–clinical risk index (age, sex, diabetes) was the strongest predictor of care demand (IRR = 1.52), exceeding the influence of individual environmental variables. Place of residence, municipality, and altitude were not significant predictors. Recruitment feasibility was high, with three refusals among 204 screened patients. Conclusions: These preliminary findings highlight the need for longitudinal, high-resolution studies and support integrating climate resilience into healthcare preparedness, early-warning systems, and sustainable health planning.

## 1. Introduction

Humankind’s ever-increasing dependence on fossil fuels and on high-input, resource-intensive agricultural practices has had profound and detrimental effects on planetary health [1]. Indeed, anthropogenic climate change is increasingly recognized as a significant threat to public health globally. Switzerland is no exception, and its climatic and geographical characteristics influence the manifestation and distribution of its health burdens [2].

Climate change contributes to the increased formation of ground-level ozone (O_3_), a major respiratory toxicant produced through photochemical reactions involving natural and anthropogenic precursors, particularly those from fossil fuel combustion. Rising temperatures, drought conditions, and other industrial emissions elevate concentrations of particulate matter (PM_2.5_ and PM_10_), composed of dust, pollen, metals, organic compounds, and, once again, combustion residues. Together with nitrous oxide (NO_2_) and sulfur dioxide (SO_2_), these pollutants constitute the most harmful airborne contaminants for human health [3]. Ambient air pollution ranks among the leading global environmental health risks, accounting for an estimated 11–12% of all deaths worldwide through pathways involving oxidative stress, systemic inflammation, and impaired immune responses. Both short-term and chronic exposure can trigger acute respiratory and cardiovascular responses, increase respiratory mortality, allergic rhinitis, lung cancer, and chronic obstructive pulmonary disease (COPD), and exacerbate asthma [4,5]. One meta-analysis found a 1.12-fold increase in COPD mortality for every 10 μg/m^3^ increase in the PM_2.5_ concentration. Similarly, for a 10 μg/m^3^ increase in the NO_2_ concentration, the relative risk (RR) of developing COPD increased by 1.6% (RR = 1.016; 95% CI: 1.012–1.120). A positive correlation has also been found between the prevalence of rhinitis and the levels of PM_10_, PM_2.5_, NO_2_, SO_2_, and O_3_ [6].

Climate change contributes to both hotter summers and colder winters, amplifying temperature-related health risks. Globally, an estimated 37% of summer heat-related deaths across 43 countries have been attributed to anthropogenic climate change [7]. Between 1983 and 2016, human exposure to extreme heat increased by 200%, affecting approximately 1.7 billion people worldwide [8]. *The Lancet* has reported that the most heat-vulnerable WHO region is Europe, with a heat vulnerability index of 40.6% recorded in 2017 [9]. Cardiovascular diseases remain the leading global cause of death, responsible for nearly 18 million deaths annually [9]. Environmental and meteorological factors, particularly temperature extremes, have been increasingly linked to elevated cardiovascular morbidity and mortality, primarily in high-income countries [10,11]. Exposure to heat induces thermoregulatory exhaustion, dehydration, hemoconcentration, and endothelial damage, which together can precipitate acute cardiac events within a short time (0–10 days) [1]. In most cases, the lag time associated with the heat effect lasts for 3–4 days [10,11]. Conversely, prolonged exposure to cold heightens sympathetic activity, causing peripheral vasoconstriction and increased myocardial oxygen demand, thereby raising blood pressure and cardiac workload [10,11]. A meta-analysis by Moghadamnia et al. (2017) [12] found that exposure to cold had a greater effect on all-cause cardiovascular disease mortality (pooled RR = 1.055; 95% CI: 1.050–1.060) than exposure to heat (pooled RR = 1.013; 95% CI: 1.011–1.015), confirming that cold remains the dominant temperature-related cardiovascular hazard.

Although climate change is a global phenomenon, Switzerland ranks among the most affected regions in Europe. According to MétéoSwiss (Switzerland’s Federal Office of Meteorology and Climatology), by 2025, national mean temperatures will have risen by 2.9 °C [2.6–3.3 °C] since the preindustrial era. This has resulted in more frequent and intense heatwaves, a 60% decline in days with frost, and an upward shift in the 0 °C isotherm of 300–400 m. The canton of Valais, identified as highly climate-sensitive, faces more frequent droughts, heatwaves, and winter storms, with a projected warming of 2.6–3.7 °C above the preindustrial era by 2060. The evolving climatic context has direct implications for healthcare [13].

Hospital emergency departments (EDs) are major points of entry into the health system, and they are already experiencing increased demand for care. Heatwaves have been associated with elevated ED use (RR = 1.06; 95% CI: 1.0–1.12). Significantly higher ED admission rates were found during heat waves compared to periods outside heat waves (RR = 1.12; 95% CI: 1.07–1.18) [14]. Switzerland has seen an 80% rise in ED admissions since the early 2000s, reaching 2.25 million outpatient visits in 2022 [15,16]. In the canton of Valais, the standardized ED use rate reached 199.4 per 1000 inhabitants in 2023, with comparable rates among women (200.5) and men (198.2) [17]

The present pilot study’s objective was to explore the preliminary effects of climate-related factors on cardiopulmonary health in the canton of Valais. Specifically, it explored the associations between daily variations in meteorological parameters (temperature and air pollution levels), patients’ demographic and clinical profiles, and the number of adult cardiopulmonary ED admissions in the canton. Within this context, the pilot study also sought to evaluate the feasibility of greater patient recruitment and broader data collection procedures in the future.

## 2. Materials and Methods

### 2.1. Study Design

Our overall mission is to adapt healthcare in the canton of Valais to its changing climatic conditions. We decided to conduct a pilot study to test the feasibility of our data collection, refine procedures, and assess the robustness of the statistical relationships generated before we implement a large-scale, canton-wide study involving longitudinal data and advanced analyses [17]. Given the inherent complexity of linking environmental exposure to health outcomes—where spatial variability, temporal lags, and data quality may introduce bias—this exploratory framework was an essential step in calibrating our methodology and generating hypotheses [17].

### 2.2. Population, Sample, and Recruitment

The study was conducted between 27 September 2024, and 20 March 2025. All patients ≥ 18 years old, presenting with cardiopulmonary comorbidities when consulting at Sion Regional Hospital’s ED, and who had been triaged at levels 3, 4, or 5 on the Valais triage and severity scale were considered eligible for participation. ICD-10 (International Classification of Diseases, Tenth Revision) diagnoses were grouped into major categories (e.g., cardiac, respiratory, infectious, digestive, neurological), enabling a clearer interpretation of admission patterns. Patients were required to be able to speak, understand, and read French and to sign an informed consent form after being made fully aware of its contents. Exclusion criteria were triage at levels 1 or 2 and a lack of decision-making capacity, as judged by an ED physician. A level-1 classification on the Valais scale indicates a life-threatening condition in which, without immediate care, the patient’s clinical status could lead to death or the loss of an organ or limb, necessitating urgent transfer to an emergency care unit. A level-2 classification requires rapid medical assessment and treatment because, although the condition is not immediately life-threatening, it has a high potential for swift deterioration. Thus, including patients triaged at levels 1 and 2 was considered inappropriate for a pilot study. Finally, Sion’s ED does not treat patients < 18 years old, and they are referred directly to the pediatric ED.

### 2.3. Procedures

The principal investigator and a trained research assistant collected data on-site following an ethics protocol approved by the Cantonal Commission for Research on Human Beings (2024-00900).

Daily meteorological and air pollution data were obtained from MétéoSwiss and RESIVAL (the canton of Valais’ ambient air pollution measurement network), including minimum, maximum, and mean daily temperatures (°C), and 24 h mean concentrations of nitrogen dioxide (NO_2_), particulate matter (PM_2.5_, PM_10_), SO_2_, and O_3_ (Table 1: Meteorological variables). PM_2.5_ and PM_10_ concentrations were obtained from distinct monitoring instruments operating under separate calibration protocols. These were matched with emergency ED admissions for adult patients (≥18 years) presenting with cardiopulmonary diagnoses at Sion’s ED between 27 September 2024, and 20 March 2025. Patient data comprised demographic (age, sex, marital status, place of residence) and clinical variables (reason for consultation, ICD-10 diagnosis, comorbidities, diabetes status, smoking status, and prior ED visits).

We collected meteorological data from different altitudes and environments, with the highest elevation at 1936 m (Chandolin, a high-mountain environment) and the lowest at 392 m (Collombey, a foothill area). According to standard European physiographic classifications, lowlands are typically situated below 500 m, mid-mountain areas are between 1000 m and 1500 m, and high-mountain regions above 1500 m [18].

We collected meteorological data comprising maximum daily temperature (Tmax), minimum daily temperature (Tmin), mean daily temperature (Tmean), and daily concentrations of O_3_, PM_10_, PM_2.5_, and NO_2_. Tmin and Tmax were only available in the data recorded at the MétéoSuisse weather station in Sion. Our other measurement locations (Montana, Saxon, and Massongex), which are part of the RESIVAL network, could only provide Tmean. Meteorological data were deterministically matched with patient ED consultation records (day 0) using a spatiotemporal linkage procedure. Each patient was assigned environmental exposure values from the monitoring station geographically closest to their place of residence on the date of their emergency department admission. When multiple stations were linked to patients on the same day, daily values were averaged. Additionally, lagged exposure variables were constructed for the ten days preceding each consultation (day 1 to day 10) to account for delayed cardiopulmonary responses to environmental conditions. This deterministic approach was selected to maximize exposure accuracy while preserving methodological feasibility within a pilot design. Finally, because altitude may influence both meteorological exposure and pollution dynamics, places of residence were categorized into three geographical zones reflecting the canton’s physiographic organization: (1) Upper Valais, corresponding to the eastern alpine region; (2) Central Valais, encompassing the mid-valley area; and (3) Lower Valais, covering the western Rhône valley [19].

Table 1 summarizes the definitions and units of measurement for all our meteorological variables.

### 2.4. Data Analysis

All data were consolidated in Excel spreadsheets (Microsoft) and analyzed using SPSS software v29.0 (IBM Corp. Armonk, NY, USA). Descriptive statistics summarized the pilot sample and meteorological variables. Because daily cardiopulmonary admissions were too infrequent for stable Poisson estimation, data were aggregated weekly to preserve temporal structure while ensuring adequate variability for inference. Aggregation is a well-established approach for enhancing variance stability and supporting more reliable statistical inference when event counts are low. This choice aligns with methodological recommendations for pilot and feasibility studies, which advocate for parsimonious analytical strategies to produce robust preliminary estimates and guide the methodological planning of subsequent large-scale investigations [20].

Weekly admission counts were modeled using Poisson regressions. Given the limited number of observations (*n* = 21) and evidence of overdispersion (φ > 1.5), a parsimonious approach was adopted, restricting models to a maximum of two predictors and applying robust sandwich estimators to obtain reliable standard errors [20,21]. Robust sandwich estimators were applied to correct standard errors, ensuring stable inference under heteroskedasticity. To capture the demographic and clinical vulnerabilities of weekly patient populations, we constructed a composite risk index using three standardized components. First, age was transformed through a logistic function to model its non-linear association with admission risk: Risk_Age = $\frac{1}{1+exp(-0.4\times \left(Age\;-60\right))}$. This function modeled risk so that it rose progressively around the inflection point of 60 years old. This normalized value (0–1) was then integrated into the composite demographic risk index. The second component was sex (1 = male, 0 = female), and the third was diabetes status (1 = present, 0 = absent). The resulting risk index was calculated as the mean of the standardized age, sex, and diabetes components. All the available sociodemographic and clinical characteristics were tested, but only these three demonstrated significant predictive value.

To prevent over-parameterization, while maximizing the analytical yield of the available data, we subsequently used a deliberately parsimonious modeling strategy [20]. Following the commonly accepted empirical guidelines for regression analysis, which require 10–15 observations per parameter, we restricted the total number of parameters to three, including the intercept [22]. Based on this constraint, three model families were constructed: one using the ten-day sine component as the base predictor, one using a ten-day cosine component, and one using the demographic–clinical risk index as its foundational term. Each meteorological variable (PM_2.5_, PM_10_, NO_2_, O_3_, and Tmean) was then introduced individually into these model families, yielding a total of 3 × 7 candidate models. Models were compared using the Akaike Information Criterion (AIC) calculated from uncorrected Poisson likelihoods to ensure internal consistency. Mean temperature was preferred over minimum and maximum temperatures to avoid multicollinearity and preserve model parsimony, given the limited number of observations.

## 3. Ethics Considerations

A formal partnership agreement between the University of Applied Sciences and Arts Western Switzerland in Valais (HES-SO Valais-Wallis) and the Valais Hospital authorized us to recruit up to 91 patients per semester, ensuring regulatory and ethics compliance. All personal data were pseudonymized in accordance with Switzerland’s Human Research Act, its General Data Protection Regulations, and the standards of the Cantonal Commission for Research on Human Beings (protocol no. 2024-00900). Data were securely stored on HES-SO Valais-Wallis’ OneDrive server, with the coding key retained by the principal investigator. Patients participated voluntarily after providing their written informed consent.

## 4. Results

This pilot study investigated the preliminary impact of climate-related factors on adult cardiopulmonary emergency admissions in the canton of Valais, Switzerland, as well as the feasibility of greater patient recruitment and broader data collection procedures in the future. Results are reported sequentially, covering patient screening and participation, participant characteristics, environmental exposures, and regression analyses examining the relationships between meteorological variables, population vulnerability, and weekly admissions.

### 4.1. Patient Participation

**Figure 1:**
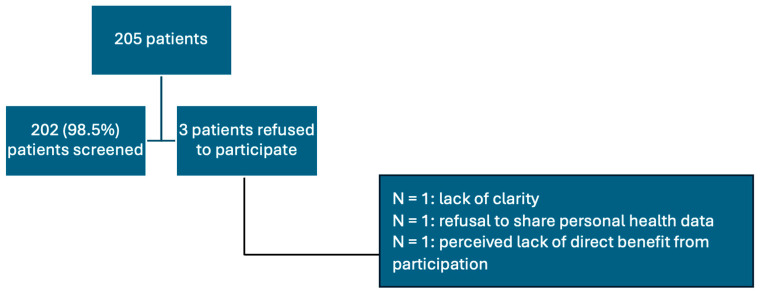
Patient screening.

During the pilot study period—27 September 2024, to 20 March 2025—202 potential participants were screened for eligibility on 90 data collection days, or 51.7% of the study’s 174-day duration. Each eligible case was reviewed prospectively and recorded on a standardized case report form that enabled consistent data acquisition across the entire observation period. A randomized subset of 91 (45%) of the 202 eligible participants was retained for analysis, corresponding to the data obtained from 50 data collection days (28.7% of study days). Randomization was performed using the Excel randomizer function. No descriptive differences were observed between the patients included and those excluded (Table 2: Participants’ sociodemographic and health data). Regarding future study feasibility, only three of the 205 patients declined to participate (Figure 1: Patient screening), indicating the recruitment method’s good feasibility and acceptability. The investigators reviewed the study protocol with both emergency care teams and patients to ensure that every procedure was clear and applicable during routine clinical practice. Subsequent data entry and analysis revealed the meteorological and sociodemographic variables to be relevant and coherent. The RESIVAL and MétéoSuisse databases used provided reliable and easily accessible measurements, reinforcing the pilot study’s robustness. Despite the limited sample size, we successfully modeled a Poisson regression, confirming the analytical and methodological feasibility of the approaches adopted in this pilot study.

### 4.2. Sociodemographic Characteristics, Health Status, and Reasons for Emergency Visits

Table 2 provides a comprehensive overview of participants’ sociodemographic characteristics, health status, and reasons for consultation, thereby contextualizing the population’s clinical vulnerability and environmental exposure. This profile is essential for interpreting subsequent analyses, as these factors may influence susceptibility to meteorological conditions and air pollution, directly supporting the study objective.

Women accounted for 58.2% of the sample’s 91 participants (mean age 67.5 ± 18.2 years; range 20–94). Half of the participants lived in central Valais (50.5%), mainly in Sion (33.0%, altitude = 512 m); thus, most were linked to Sion’s RESIVAL weather monitoring station (69.2%) Most of the study sample lived in foothill areas (67%), with smaller proportions in lowland zones (16%) and mid-mountain regions (11%). Only a few participants (about 6%) lived in high-mountain environments, indicating that the sample largely represented settlements situated at moderate altitudes. Over half of the participants were married (54.9%).

The predominant reasons for an ED consultation were respiratory distress or dyspnea (34.1%) and chest or cardiovascular pain (34.1%). Cardiovascular comorbidities were frequent (71.4%), including hypertension or orthostatic hypotension (35.5%), arrhythmias (26.2%), ischemic heart disease (26.2%), and heart failure (13.8%). Respiratory conditions were also common (40.7%), particularly COPD (37.8%) and sleep apnea (37.8%). Active smoking was reported by 42.9% of participants (mean = 33.3 pack-years ± 28.4), and over half (52.7%) had at least one additional comorbidity, notably genitourinary (33.3%), metabolic or endocrine (33.3%), oncological (29.2%), and hepatobiliary or digestive (25.0%) disorders. Most patients were not diabetic (80.2%), and 35.2% had been admitted to the ED within the preceding six months.

When comparing seasonal data, patients’ demographic and clinical profiles were largely similar, though some seasonal variations emerged. Participants were slightly older in autumn (68.7 vs. 66.9 years), dyspnea and respiratory disorders were more prevalent in winter (37.7% vs. 26.7%), whereas thoracic or cardiovascular pain were balanced (33.3% vs. 34.4%) (Table 2: Participants’ sociodemographic and health data).

Table 2 characterizes a population with substantial cardiopulmonary vulnerability and relevant environmental exposure, providing essential context for interpreting the associations between climate-related factors and emergency admissions.

### 4.3. Descriptive, Associative, and Inferential Analysis of the Collected Data

#### 4.3.1. Data Aggregation

**Figure 2:**
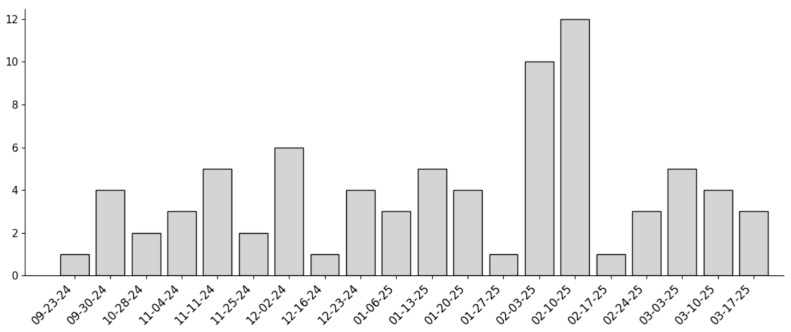
Number of weekly admissions.

The analysis covered 21 consecutive weeks. Weekly admission counts ranged from 1 to 14, with a mean of 4.24. Most weeks recorded between 1 and 6 admissions; however, there were two peak weeks in late January and early February (with 11 and 14 admissions, respectively). Low-count weeks (1–2 admissions) represented 28.6% of the period, and moderate-count weeks (3–5 admissions) made up 42.9% (Figure 2: Number of weekly admissions).

#### 4.3.2. Longitudinal Description of the Evolution of Atmospheric Pollutants and Temperatures

Aligned with the study objective of assessing the health relevance of climate-related exposures, a longitudinal descriptive analysis of temperature and atmospheric pollutant patterns throughout the study period was performed. Examining these temporal variations is critical for contextualizing environmental exposure and for informing the interpretation of subsequent models evaluating their associations with cardiopulmonary emergency admissions. This descriptive step, therefore, provides the environmental basis for understanding how short-term fluctuations in meteorological conditions may relate to observed healthcare utilization.

**Figure 3:**
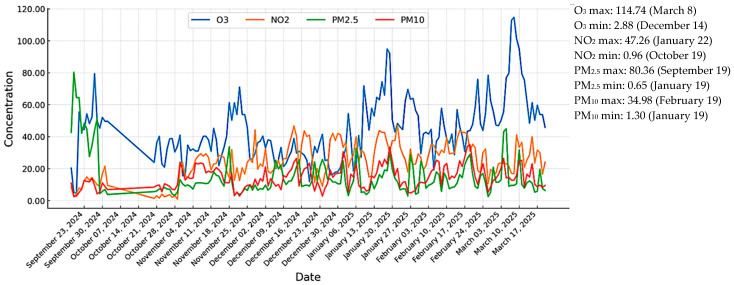
Daily evolution of atmospheric pollutants.

Figure 3 illustrates the daily evolution of the main atmospheric pollutants—O_3_, PM_10_, PM_2.5_, and NO_2_—incorporating same-day (T_0_) and lagged exposures up to ten days (T_10_). Characterizing these temporal patterns is essential for understanding environmentally driven variability in cardiopulmonary risk. Overall, the pollutants displayed marked seasonal dynamics shaped by meteorological conditions and emission sources.

O_3_ concentrations peaked at 114.7 µg/m^3^ on 8 March 2025, consistent with enhanced photochemical formation under sunnier early-spring conditions, whereas the minimum value (2.9 µg/m^3^) occurred on 14 December 2024, during a cold and stable atmospheric period. In contrast, PM exhibited higher concentrations during autumn and winter, when domestic heating and stagnant air masses typically intensify emissions. PM_2.5_ reached 80.4 µg/m^3^ on 19 September 2024, suggesting early seasonal accumulation, while PM_10_ peaked at 35.0 µg/m^3^ on 19 February 2025, coinciding with a late-winter inversion episode. The lowest PM_2.5_ (0.6 µg/m^3^) and PM_10_ (1.3 µg/m^3^) values were recorded on 19 January 2025, when strong winds likely enhanced particulate dispersion. NO_2_ followed a comparable cold-season pattern, with a maximum of 47.3 µg/m^3^ on 22 January 2025, and a minimum of 0.9 µg/m^3^ on 29 October 2024, reflecting the influence of traffic and heating emissions under limited atmospheric mixing.

**Figure 4:**
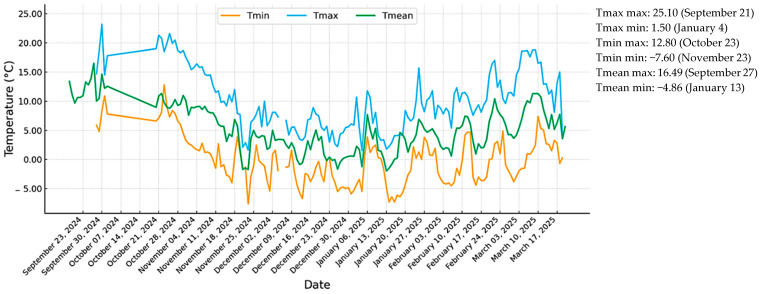
Daily changes in temperature.

Figure 4 illustrates the evolution of Tmax, Tmean, and Tmin over time, as recorded between 18 September 2024, and 20 March 2025, integrating same-day (T_0_) and lagged (T_1_–T_10_) observations. During the pilot study’s early period, temperatures were relatively high, with a pronounced peak of 25.1 °C recorded on 21 September 2024. Thereafter, Tmax gradually and continuously declined as autumn progressed, reaching its lowest value of 1.5 °C on 4 January 2025. This transition marks the onset of winter conditions and reflects a clear loss of the sun’s daytime heating capacity during the cold season. The highest Tmin value (12.8 °C) was observed on 23 October 2024, indicating the mild nighttime conditions typical of early autumn. Conversely, the lowest Tmin (−7.6 °C) occurred on 23 November 2024, revealing strong nocturnal radiative cooling and winter’s early onset. The amplitude between daily maximums and minimums demonstrated substantial thermal variability and the progressive establishment of colder masses of air as the season advanced. The highest Tmean value (16.49 °C) was recorded on 27 September 2024, while the lowest (−4.86 °C) occurred on 13 January 2025. The September to January decline in Tmean reflected the cumulative seasonal cooling trend, and this was followed by a gradual recovery from late February onward, consistent with the beginning of springtime warming. Overall, the three variables revealed a marked seasonal thermal cycle, characterized by a progressive cooling phase from early autumn to midwinter and a subsequent moderate warming trend toward early spring. The clear synchronicity between Tmax, Tmin, and Tmean confirmed the data’s internal consistency. It provided a robust meteorological representation of the transition from warm to cold seasons in a temperate climate.

#### 4.3.3. Regression Analysis

Across the 21 candidate models, several predictors improved model fit relative to their respective baselines. Within the cosine family (Table 3), both O_3_ and mean weekly temperature enhanced performance, with temperature producing the largest improvement (ΔAIC = 3.7 versus ΔAIC = 1.7). In the sine family (Table 4), the demographic–clinical risk index provided the strongest gain in predictive performance (ΔAIC = 7.9). These rankings were consistent with quasi-log-likelihood comparisons. Table 3 and Table 4 present the full set of Poisson regression models used to examine weekly cardiopulmonary emergency admissions. Table 3 summarizes models including the cosine periodic component, whereas Table 4 reports models including the sine periodic component as the base temporal predictor. For each model, regression coefficients (β) are shown with robust standard errors in parentheses, together with model fit indicators (N, AIC, BIC, log-likelihood, and dispersion parameter φ). Abbreviations are as follows: 10_day_cos and 10_day_sin denote the cosine and sine components of the ten-day periodic cycle; Risk_mean refers to the weekly demographic–clinical risk index; O_3__mean, PM_10__mean, PM_2.5__mean, and N_O2__mean indicate weekly mean pollutant concentrations; and Tmean represents the weekly mean temperature.

Based on AIC, three models emerged as the most parsimonious and robust: (1) cos_Tmean (AIC = 100.4), (2) cos_O_3_ (AIC = 102.5), and (3) sin_risk (AIC = 102.6). The best-performing model, cos_Tmean, included the ten-day cosine component (β = −0.481, SE = 0.188, *p* < 0.05) and mean weekly temperature (β = −0.080, SE = 0.046, *p* < 0.1). The second model, cos_O_3_, combined the periodic cosine term (β = −0.484, SE = 0.191, *p* < 0.05) with mean O_3_ concentration (β = 0.014, SE = 0.008, *p* < 0.1). The third model, sin_risk, incorporated the sine component (β = 0.396, SE = 0.289, *p* = 0.1) together with the demographic–clinical risk index (β = 2.315, SE = 0.776, *p* < 0.01), corresponding to a substantial standardized IRR of 1.52. Overall, the cosine term appeared in two of the three best models, supporting its central role in explaining week-to-week variation in cardiopulmonary emergency admissions. Temperature and O_3_ contributed to additional improvements in cosine-based models, whereas the risk index substantially strengthened the sine-based specification. Together, these findings suggest that seasonal periodicity, short-term meteorological and pollution variations, and demographic–clinical vulnerability may all influence weekly cardiopulmonary admissions, with the cosine–temperature model providing the best fit in this pilot dataset. In contrast, PM_10_ (β = −0.041, SE = 0.038), PM_2.5_ (β = −0.005, SE = 0.030), and NO_2_ (β = −0.0011, SE = 0.018) showed effect estimates close to null and were not statistically significant, indicating that no measurable short-term associations with weekly cardiopulmonary admissions were detected for these pollutants in the present pilot study.

## 5. Discussion

Our pilot study identified four primary determinants of weekly cardiopulmonary ED admissions: (1) demographic–clinical vulnerability (age, sex, and diabetes), (2) mean weekly temperature, (3) O_3_ concentrations, and (4) a ten-day periodic cycle. Together, these findings suggest that short-term healthcare demand in an alpine setting may be shaped by the interaction between environmental exposures and population susceptibility. Integrating these dimensions into predictive frameworks could therefore support more anticipatory and climate-resilient healthcare planning.

We observed that Tmean was inversely associated with weekly admissions. Indeed, the negative temperature coefficient corresponded to an incidence rate ratio (IRR) of 0.92, indicating that a one-unit increase in weekly mean temperature was associated with an 8% reduction in expected weekly cardiopulmonary ED admissions, after adjusting for periodicity. Thus, colder weeks were associated with higher admission counts. This negative association could indicate that colder periods were linked to more frequent consultations. This result is consistent with evidence showing that cold exposure induces physiological stress through mechanisms such as sympathetic activation, vasoconstriction, and increased cardiac workload, thereby elevating cardiovascular and respiratory risk. The coincidence between the coldest recorded period and peaks in ED admissions further reinforces the plausibility of this relationship. The 2017 meta-analysis by Moghadamnia et al. [12] demonstrated that exposure to low temperatures increased all-cause cardiovascular mortality more than exposure to heat (RR = 1.055 vs. 1.013). Similarly, recent European evidence [23] showed that extreme cold events contributed disproportionately to winter mortality in both temperate and alpine climates. In our data, the coldest day (23 January 2025: Tmin = −4.8 °C) coincided with our two peaks in weekly admissions, reinforcing evidence for this physiological link. By contrast, even though the temperature series documented a pronounced heat episode in July 2024 (Tmax = 36.5 °C), the absence of a detectable heat effect likely reflects the pilot study’s seasonal scope and limited statistical power rather than the absence of a true association, as prior research has documented increased emergency visits during heatwaves. Taken together, these findings underscore the importance of considering cold-related risk alongside heat preparedness in alpine health system planning. A 2021 study by Bujosa Mateu et al. (2024) [14] reported increases in total ED visits during heatwaves (RR = 1.12; 95% CI: 1.07–1.18), as did a 2015 systematic review, which observed a 2–11% rise in hospital and ED admissions during heatwaves [24].

The incidence rate ratio calculated for O_3_ (IRR = 1.014) indicated that short-term increases in this air pollutant were associated with modest yet consistent rises in weekly cardiopulmonary admissions. This finding aligns with established evidence showing that acute O_3_ exposure exacerbates respiratory and cardiovascular conditions, including the risk of asthma, arrhythmias, and ischemic events, particularly under the warm, sunny meteorological conditions that favor photochemical O_3_ formation [25]. Conversely, the effects of PM_10_, PM_2.5_, and NO_2_ pollution did not reach statistically significant levels. Given the exploratory design and small sample size, these findings should be interpreted cautiously, as multicollinearity and limited temporal variability may have attenuated observable effects. The literature has clearly demonstrated their effects on cardiopulmonary complications. Each 10 µg/m^3^ rise in PM_2.5_ has been associated with roughly a 1.12-fold increase in COPD mortality, and each 10 µg/m^3^ rise in NO_2_ with a 1.6% increase in chronic respiratory risk [25,26]. Our data captured winter peaks in NO_2_ and PM_10_ (19 December and 19 February 2025, respectively) that matched the seasonal accumulation of combustion emissions typical of cold, stagnant air masses in alpine valleys.

Finally, the sociodemographic and contextual determinants of age, sex, and diabetes status constituted a joint risk index that was associated with a 52% increase in expected weekly ED admissions. The literature highlights that health-related factors, including sex, age, and the presence of chronic comorbidities, significantly modulate vulnerability to weather-related effects on health [9]. According to a 2022 meta-analysis by Liu et al. [27], the relative risk of temperature-related morbidity among individuals aged ≥65 years, compared to those younger, is higher by 0.8% for each 1 °C rise in temperature (1.7%; RR = 1.017 [95% CI: 1.016–1.019] vs. 0.9%; RR = 1.009 [95% CI: 1.004–1.014]; *p* = 0.03). In terms of sex-specific responses, evidence indicates that men are generally more susceptible to cold-related cardiovascular effects, while women—particularly older adult women—exhibit greater vulnerability to heat-related outcomes. Beyond biological sex, social determinants such as marital status also appear to influence weather-related health risks: unmarried individuals demonstrate a higher risk of adverse outcomes during extreme heat than their married counterparts, even after controlling for age [28].

Beyond individual demographics, local environmental features are known to modulate exposure and vulnerability [29]. The Valais region’s topography favors temperature inversions and pollutant trapping, while the valley floor in which Sion finds itself concentrates both traffic and residential heating emissions. International evidence underscores that urban areas and reduced green space increase vulnerabilities to thermal extremes and exposure to pollution. With Switzerland’s urban green coverage estimated at 32.35%, expanding vegetated buffer zones around residential areas could mitigate local exposure risks. Indeed, European data attributes approximately 43,000 preventable premature deaths annually to proximity to green spaces [26]. Given the data we collected, none of the models tested found place of residence, municipality, and altitude to be significant predictors of cardiopulmonary risk in the canton of Valais.

This pilot study offers important conceptual insights by demonstrating the feasibility of integrating real-time environmental indicators with clinical utilization data. Rather than acting as isolated drivers, meteorological conditions, air quality, and demographic vulnerability appear to function as interconnected determinants of healthcare demand. This integrative perspective represents a meaningful step toward developing predictive models capable of supporting proactive resource allocation under changing climatic conditions.

### 5.1. Recommendations for Research

Following this pilot study, we believe that strengthening our understanding of the weather-related health mechanisms in the canton of Valais is indeed feasible. Integrating available sociodemographic variables and real-time meteorological–clinical data should be linked to qualitative insights into patient adaptation. Patients’ willingness to participate suggests that future research should employ multi-site, longitudinal designs and collect higher-resolution clinical and environmental data. Supported by advanced modeling approaches, such as Distributed Lag Non-Linear Models or Generalized Additive Models, this would better capture interannual variability and extreme events. However, developing a reliable and operational predictive model will require higher-frequency data: robust estimation generally demands 40–50 observations per parameter. For even minimally parameterized models, this translates into approximately 250–300 observations, which would only be achievable through near-daily data collection. Future studies, with richer, non-aggregated datasets, will therefore be essential to produce deployable prediction tools capable of supporting dynamic hospital capacity management.

### 5.2. Recommendations for Teaching

Nursing has long recognized the intrinsic link between the environment and health, a principle rooted in Florence Nightingale’s philosophy of creating the optimal conditions for nature to promote healing. In the era of climate change, integrating environmental and weather-related factors into nursing education and practice will be vital to helping professionals anticipate, prevent, and manage those factors’ health effects. To ensure the effectiveness and added value of such educational initiatives, nurses’ levels of eco-literacy must be systematically assessed. This will allow programs to be tailored to nursing students’ needs and their impact on knowledge, attitudes, and clinical practice to be evaluated objectively.

### 5.3. Recommendations for Practice

With a changing climate, healthcare systems must strengthen their preparedness for weather-related health risks by developing contingency plans for heat and cold waves, integrating real-time surveillance of temperature, air pollution, and hospital admission patterns, and adopting sustainable, low-carbon models of care. Targeted prevention should prioritize vulnerable groups—older adults, those with chronic diseases, and socioeconomically disadvantaged populations—who face heightened environmental health risks.

### 5.4. Strengths and Limitations

This pilot study, based on 91 patients spread over 21 weekly observations, was exploratory in nature. The limited temporal resolution and small sample size reduced statistical sensitivity to short-term fluctuations, which may account for the absence of detectable effects due to concentrations of PM_10_, PM_2.5_, and NO_2_ that are typically reported in larger epidemiological studies. Although weekly aggregation constrained individual-level interpretability and attenuated short-cycle dynamics, the study demonstrated several strengths, such as the feasibility of integrating environmental and clinical data streams in near real time. Then, the identification of ten-day cyclical patterns is consistent with known lag structures in cardiopulmonary responses. More broadly, the findings underscored the seasonal dual vulnerabilities facing alpine populations—winter cold and pollution events, and summer ozone peaks—which have implications for climate-resilient healthcare planning.

## 6. Conclusions

One of the major challenges facing healthcare is the need to address health inequities and ensure that vulnerable populations are adequately protected from the effects of changing weather patterns on health. In line with the United Nations Sustainable Development Goals, specifically Goal 3 (Good Health and Well-being) and Goal 13 (Climate Action), urgent action is required to strengthen healthcare systems’ resilience and capacity to adapt to climate-related hazards. Transitioning from a reactive care system to a proactive health system requires reorienting resources toward prevention, health promotion, and sustainability. With this future in mind, strengthening predictive approaches that incorporate meteorological exposure and patient vulnerability could support more proactive, climate-resilient health system planning. This pilot study explored the preliminary associations between climate-related factors and adult cardiopulmonary emergency admissions in the canton of Valais, Switzerland. The findings suggest that weekly mean temperature, ozone concentrations, demographic–clinical vulnerability, and short-term periodic dynamics may contribute to fluctuations in admission patterns. Although place of residence, municipality, and altitude were not significant predictors, the results highlight the relevance of integrating environmental indicators with population risk profiles to better anticipate climate-sensitive healthcare demand. Importantly, this pilot study also successfully informed the planning of a future full-scale study by confirming the feasibility of patient recruitment, data linkage procedures, and analytical strategies, while clarifying sample size requirements and key methodological parameters needed for robust inference.

## 7. Patients

Patients contributed to this study during their emergency department visits by providing clinical and demographic data that enabled us to link environmental exposure with their health information. They were not involved in study design, analysis, or manuscript preparation.

## Figures and Tables

**Table 1:** Meteorological variables.

Variable	Description	Unit	Source
Tmax	Daily maximum air temperature measured at ~2 m above ground level	°C	MétéoSuisse (Sion weather station only)
Tmin	Daily minimum air temperature measured at ~2 m above ground level		
Tmean	Daily mean air temperature measured at ~2 m above ground level		RESIVAL (Les Giettes, Massongex, Sion, Saxon, Montana)
O_3_	Daily average concentration of ozone	µg/m^3^	RESIVAL (Les Giettes, Massongex, Sion, Saxon, Montana)
PM_10_	Daily average concentration of particulate matter ≤ 10 µm		
PM_2.5_	Daily average concentration of particulate matter ≤ 2.5 µm		
NO_2_	Daily average concentration of nitrogen dioxide		

**Table 2:** Participants’ sociodemographic and health data.

	Autumn (27 September 2024–20 December 2024) (*n* = 30)	Winter (21 December 2024–20 March 2025) (*n* = 61)	Total
Sex, *n* (%)			
Male	13 (43.3%)	25 (41.0%)	38 (41.8%)
Female	17 (56.7%)	36 (59.0%)	91 (58.2%)
Areas of Valais, *n* (%)			
Central Valais	17 (85.0%)	29 (82.9%)	46 (50.5%)
Upper Valais	0 (0%)	1 (2.9%)	1 (1.1%)
Lower Valais	3 (15.0%)	5 (14.3%)	8 (8.8%)
RESIVAL weather station, *n* (%)			
Sion	25 (83.3%)	38 (62.3%)	63 (69.2%)
Saxon	3 (10.0%)	11 (18.0%)	14 (15.4%)
Montana	2 (6.7%)	11 (18.0%)	13 (14.3%)
Massongex	0 (0%)	1 (1.6%)	1 (1.1%)
Marital status, *n* (%)			
Married	14 (46.7%)	36 (59.0%)	50 (54.9%)
Single	6 (20.0%)	11 (18.0%)	17 (18.7%)
Divorced	6 (20.0%)	5 (8.2%)	11 (12.1%)
Widowed	4 (13.3%)	9 (14.8%)	13 (14.3%)
Reason for consultation, *n* (%)			
Dyspnea/Respiratory disorders	8 (26.7%)	23 (37.7%)	31 (34.1%)
Thoracic/Cardiovascular pain	10 (33.3%)	21 (34.4%)	31 (34.1%)
Abdominal/Digestive pain	2 6.7%)	3 (4.9%)	5 (5.5%)
Neurological	5 (16.7%)	11 (18.0%)	16 (17.6%)
Trauma/Fractures	1 (3.3%)	1 (1.6%)	2 (2.2%)
Urinary retention	0 (0%)	1 (1.6%)	1 (1.1%)
Hemorrhages	3 (10.0%)	1 (1.6%)	4 (4.4%)
Skin infection/other diagnoses	1 (3.3%)	0 (0%)	1 (1.1%)
Medical and surgical history			
Cardiac history, *n* (%)	21 (70%)	44 (71.2%)	65 (71.4%)
Blood pressure disorders	9 (42.9%)	14 (31.8%)	23 (35.5%)
Dyslipidemia	4 (19.0%)	7 (15.9%)	11 (16.9%)
Cardiopathy	19 (90.5%)	17 (38.6%)	36 (55.4%)
Heart failure	6 (28.6%)	3 (6.8%)	9 (13.8%)
Thromboembolic disorders	8 (38.1%	7 (15.9%)	10 (15.4%)
Heart transplant	1 (4.8%)	0 (0%)	1 (1.5%)
Respiratory history, *n* (%)	14 (46.7%)	23 (37.7%)	37 (40.7%)
COPD	9 (64.3%)	5 (21.7%)	14 (37.8%)
OSAS	4 (28.6)	6 (26.1%)	10 (27.0%)
Allergic respiratory disease	3 (21.4%)	3 (13.0%)	6 (16.2%)
Thromboembolic events	0 (0%)	5 (21.7%)	5 (5.5%)
Infectious respiratory diseases	1 (7.1%)	4 (17.4%)	5 (5.5%)
Airway structural disorders	1 (7.1%)	3 (13.0%)	4 (4.4%)
Smoking status, *n* (%)			
Yes	16 (53.3%)	23 (37.7%)	39 (42.9%)
No	14 (46.7%)	38 (62.3%)	52 (57.1%)
Former smoker	14 (46.7%)	13 (21.3%)	27 (29.7%)
Diabetes status, *n* (%)			
Yes	4 (13.3%	14 (23.0%)	18 (19.8%)
No	26 (86.7%)	47 (77.0%)	73 (80.2%)
Comorbidities, *n* (%)			
Yes	13 (43.3%)	35 (57.4%)	48 (52.7%)
Hepatobiliary/Digestive	2 (15.4%)	10 (28.6%)	12 (25.0%)
Vascular	2 (15.4%)	4 (11.4%)	6 (12.5%)
Metabolic/Endocrine	4 (30.8%)	12 (34.3%)	16 (33.3%)
Oncological	3 (23.1%)	11 (31.4%)	14 (29.2%)
Genitourinary	4 (30.8%)	12 (34.3%)	16 (33.3%)
Musculoskeletal/Orthopedic	3 (23.1%)	7 (20.0%)	10 (20.8%)
Neurological	3 (23.1%)	4 (11.4%)	7 (14.6%)
Psychiatric	0 (0%)	2 (5.7%)	2 (4.2%)
Hematological/Allergies/Immunological	1(7.7%)	3 (8.6%)	4 (8.4%)
No	17 (56.7%)	26 (42.6%)	43 (47.3%)
ED readmissions, *n* (%)			
Yes	11 (36.7%)	21 (34.4%)	32 (35.2%)
No	19 (63.3%)	40 (65.6%)	59 (64.8%)
Age [years], mean (SD)	68.7 (18.2)	66.9 (18.5)	67.5 (18.2)
Age [years], min–max	20–91	25–94	20–94
Pack-years, mean (SD)	33.8 (32.6)	32.9 (23.5)	33.3 (28.4)
Pack-years, min–max	1–100	1–82.5	1–100

**Table 3:** Weekly admission model comparison—baseline model, COS periodic term.

	Base_ Cos	Cos_ Risk	Cos_ O_3_	Cos-PM_10_	Cos_ PM_2.5_	Cos_ NO_2_	Cos_ Tmean
Intercept	1.407 *** (0.160)	0.855 ** (0.361)	0.975 *** (0.302)	1.713 *** (0.393)	1.434 *** (0.323)	1.583 *** (0.327)	1.777 *** (0.301)
10_day_cos	−0.436 ** (0.180)	−0.334 * (0.172)	−0.484 ** (0.191)	−0.411 ** (0.199)	−0.434 ** (0.195)	−0.441 ** (0.185)	−0.481 ** (0.188)
Risk_mean		1.195 (0.766)					
O_3__mean			0.014 * (0.008)				
PM_10__mean				−0.023 (0.029)			
PM_2.5__mean					−0.002 (0.018)		
NO_2__mean						−0.008 (0.013)	
Tmean							−0.080 * (0.046)
N	21	21	21	21	21	21	21
AIC	104.1	103.8	102.4	104.7	106.1	105.1	100.4
BIC	106.2	106.9	105.6	107.8	109.3	108.8	103.5
Log-Likelihood	−50.072	−48.886	−48.224	−49.331	−50.060	−49.856	−47.183
Dispersion	2.103	2.050	2.020	2.209	2.212	2.210	1.714

* *p* < 0.1; ** *p* < 0.05; *** *p* < 0.01.

**Table 4:** Weekly admission model comparison—baseline model, SIN periodic term.

	Base_ Sin	Sin_ Risk	Sin_ O_3_	Sin_ PM_10_	Sin_ PM_2.5_	Sin_ NO_2_	Sin_ Tmean
Intercept	1.438 *** (0.177)	0.328 (0.371)	1.110 *** (0.290)	1.963 *** (0.577)	1.508 *** (0.416)	1.682 *** (0.497)	1.689 *** (0.380)
10_day_sin	0.275 (0.292)	0.396 (0.289)	0.276 (0.283)	0.366 (0.273)	0.265 (0.285)	0.313 (0.293)	0.164 (0.312)
Risk_mean		2.315 *** (0.766)					
O_3__mean			0.011 (0.008)				
PM_10__mean				−0.041 (0.038)			
PM_2.5__mean					−0.005 (0.030)		
NO_2__mean						−0.0011 (0.018)	
Tmean							−0.051 (0.0453)
N	21	21	21	21	21	21	21
AIC	110.5	102.6	110.1	108.2	112.4	111.7	110.4
BIC	112.6	105.7	113.3	111.3	115.5	114.8	113.5
Log-Likelihood	−53.272	−48.292	−52.066	−51.084	−53.182	−52.857	−52.188
Dispersion	2.455	1.965	2.462	2.203	2.600	2.488	2.325

*** *p* < 0.01.

## Data Availability

No data available due to ethical restrictions.
